# Ophthalmia Neonatorum

**Published:** 1891-10-24

**Authors:** 


### OPHTHALMOLOGY.

### OPHTHALMIA NEONATORUM.

The fact that a large number of eyes are annually lost by this complaint makes its study more than usually interesting. Of the eyes that go blind ten per cent. can be attributed to this disease. Two or three days after birth the lids appear swollen or puffy, and the margins are stuck together. Upon separating the lids the conjunctiva is found to be very vascular, and soon a purulent secretion commences. It is generally at a later date that the ophthalmic surgeon first sees it. He then ascertains the date of commencement, as unless it be within six days from the birth, the infection is probably not derived from the vaginal secretions. Having placed the child's head between his (the surgeon's knees), he separates the lids, and finds the purulent secretion emanating chiefly from the palpebral conjunctivæ, which appear vascular and velvety, and readily bleed when touched. Removing the discharge with dossils of lint, he proceeds to examine the cornea, which, if uninjured, appears bright and transparent, but if affected dull like the eye of a dead fish, or, may be, he finds it actually ruptured.

Should there be any difficulty in opening the lids, retractors should be used, or the spring speculum, but if from swelling of the lid this be impossible, the upper lid may be slit vertically, and the flaps turned back to expose the eyeball. When there is so much swelling (chemosis), the cornea appears sunk, and is often considerably obscured by the surrounding elevated conjunctiva. The cause of this disease is contact during birth with vaginal secretions or just after birth from carelessness before the child has been washed. It is generally considered that the vaginal secretions must be unhealthy, that gonorrhœa, leucorrhœa, or some allied disease must exist in the mother. In treatment, we must consider both modes of (1) prevention and (2) cure. As regards prevention, absolute cleanliness must be observed. Should any vaginal discharge exist, antiseptic injections should be given at short intervals during labour; this is easily done, without annoying the patient, by means of the syphon douche. The doctor and other attendants should on no account open the eyelids until after the child has been washed and their own hands thoroughly cleansed, when, should the mother be known to be suffering from any infectious discharge, or even should a suspicion of such exist, a lotion of nitrate of silver (2 per cent.) should be dropped into the conjunctival sacs. Credé goes so far as to drop it into the eyes of every child, whether or no the mother has a vaginal discharge; but this seems unnecessarily severe, as anyone will decide should he drop a 2 per cent. solution of nitrate of silver into his own eye.

Should no symptoms show themselves within six days, the child may be considered secure, as far as infection from vaginal secretions is concerned. When the disease reaches the stage just now described, as the most usual for the ophthalmic surgeon to first see it in, the palpebral conjunctivæ should be painted over with a solution of nitrate of silver, 10 grains or 20 grains to the ounce of water, and the nurse be directed to irrigate the eye by washing with luke-warm water squeezed from a clean new sponge, and then to apply an antiseptic lotion, this process to be repeated hourly, night and day. The lotion may consist of nitrate of silver 1-2 grains to the ounce, hydrarg. perchlor. 1 in 5,000, boroglyceride 20 per cent., thallin sulph. 1 per cent., dilute Condy's fluid, &c., the painting to be repeated by the surgeon at the end of 24 hours. As the eye improves, astringents may be used, alum sulph., zinci-sulphocarbol, zinci sulph., cupri sulph., &c., 1-2 grains to the ounce of water. Ulceration should be treated by quinine or eserin lotions, in addition to the irrigation as above.

Cases seen before the cornea is affected, and vigorously treated, have a good chance of recovery, but if treatment be neglected the results are, as a rule, very bad. It is, therefore, most important to impress upon patients the gravity of a "cold in the eyes" in the new-born babe, and the fearful consequences of neglect of such.

The results are, when treatment is neglected—(1) Recovery in a few cases; (2) opacities of the cornea from ulceration and subsequent cicatrisation; (3) sloughing of the cornea (the vessels supplying the cornea being strangulated by the chemosed conjunctiva), followed by loss of the lens and vitreous; (4) prolapse of iris through peripheral ulcer; (5) anterior staphyloma, from the cicatrix of a healed ulcer bulging out under increased tension of the ocular fluids; (6) pyramidal cataract. A central ulcer perforating allows the aqueous humour to escape and the lens is pushed forward, lymph forms on the exposed surfaces of the lens capsule, and the aqueous humour being again secreted presses the lens back again with the attached lymph, and the ulcer heals, leaving the lens thus damaged.

The further treatment of these disastrous results does not come within the limits of this paper, but when we look at the list, and remember how many go blind annually from neglect of this disease, we recognise the importance of early and efficient treatment, and, what is more, of prevention of the disease.

